# A Case of Disseminated Hypopigmented Keratoses Improved with Oral Acitretin

**DOI:** 10.1155/2017/1617375

**Published:** 2017-12-21

**Authors:** Ali Haydar Eskiocak, Cumhur İbrahim Bassorgun, Sarp Uzun, Soner Uzun

**Affiliations:** ^1^Department of Dermatology and Venereology, Akdeniz University School of Medicine Hospital, Antalya, Turkey; ^2^Department of Pathology, Akdeniz University School of Medicine Hospital, Antalya, Turkey; ^3^Hacettepe University School of Medicine, Ankara, Turkey

## Abstract

Disseminated hypopigmented keratosis is a distinct clinical entity and only few cases have been reported so far. Here, we present a 21-year-old man with almost 10-year history of hypopigmented, nonfollicular, keratotic lichenoid papules occurring on the extensor surfaces of the extremities, back and lumber region. Histopathological examination showed orthohyperkeratosis, irregular acanthosis, and sporadic papillomatosis with a normal amount of melanin and number of melanocytes. In addition, no marked inflammation or melanophages were seen. In order to exclude other possible causes, we performed laboratory tests and radiological examination which were all found to be normal. As the clinical and histopathological features of our patient were taken into account, it was considered to be compatible with the diagnosis of disseminated hypopigmented keratoses. So far, only topical therapies have been used with failure in the previously reported cases except one patient. Considering the extensive lesions, we treated the present patient with 5% salicylic acid in addition to oral acitretin and significant regression in all lesions was achieved, particularly on the keratosis.

## 1. Introduction

Disseminated hypopigmented keratosis (DHK) was first described by Morison et al. in 1991 [1]. No case was reported till the study with the 13 cases of hypopigmented keratosis (HK) was reported by Kim et al. in 2013 [2]. Here we report a case presented with diffuse hypopigmented keratotic lesions considered with the diagnosis of DHK which was improved by oral acitretin and we compared the characteristics of our case with the cases reported before.

## 2. Case Report

A 21-year-old man referred to our outpatient clinic because of the numerous asymptomatic hypopigmented lesions which appeared 10 years ago. The lesions persisted since the beginning and they had a course of gradual and slow increase in number. The lesions were disseminated during this period. He had no history of any systemic disease or phototherapy/UV exposure and he was healthy in all other respects. The family history was unremarkable.

**Figure 1:**
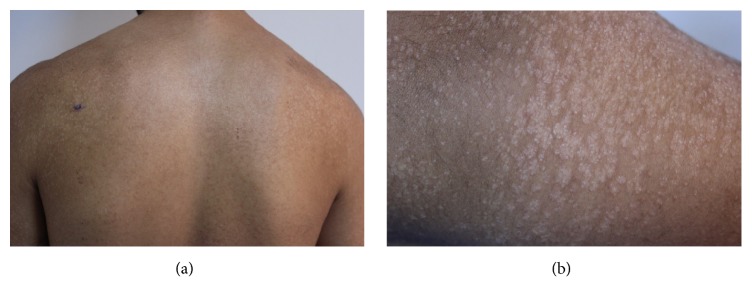
(a) The hypopigmented keratotic lesions on the upper back. (b) The close-up appearance of the lesions on the extensor surface of the right upper arm.

**Figure 2:**
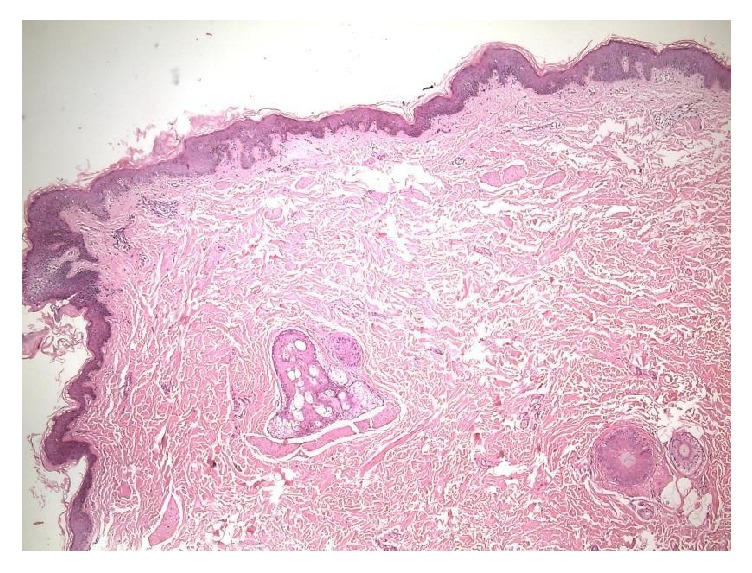
Orthohyperkeratosis, irregular acanthosis, and sporadic papillomatosis. HE ×40 (HE: hematoxylin and eosin).

Dermatological examination revealed hypopigmented, 2–5 mm in size, nonfollicular, numerous keratotic lichenoid papules on the whole body with a symmetrical distribution (Figure 1(a)). The lesions coalesced to form larger and linear lesions on the extensor surfaces of the upper arms (Figure 1(b)). Multiple direct mycological examinations and fungal culture were studied from the scales which were all unremarkable. Histopathological examination of the lesions revealed orthohyperkeratosis, irregular acanthosis, and sporadic papillomatosis (Figure 2). Routine laboratory examination including complete blood count, renal and liver function tests, and chest radiography were all found to be normal.

**Figure 3:**
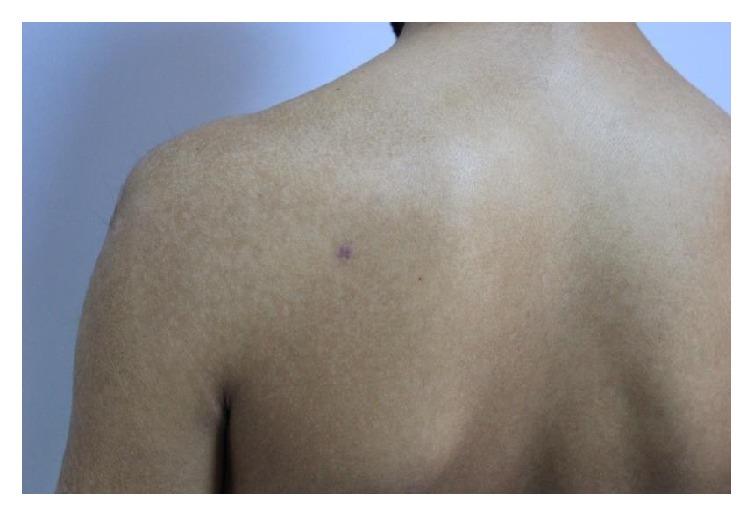
The appearance of the left upper back and the left upper arm after 2 months of the treatment.

The patient was treated with topical 5% salicylic acid in petrolatum for 2 weeks and oral acitretin for 2 months and showed significant improvement predominantly on the keratosis (Figure 3). No major side effect was seen related to the use of acitretin.

## 3. Discussion

Here we presented a case of DHK improved with oral acitretin that represents a distinct entity with a unique presentation [1]. The reported cases of DHK are listed and summarized in Table 1. In 2002, Kokturk et al. reported a localized variant of this entity presented in a 16-year-old girl [3].

In the study reported by Kim et al., 13 cases of HK were reported and 4 of them had a disseminated distribution [2]. The authors suggested that HK may be a “hyperkeratotic” variant of idiopathic guttate hypomelanosis [2]. The lesions are distributed more intensively and extensively in our patient compared to previous reported cases. Additionally, the papules coalesced in some areas and were more scaly in our patient. Therefore it could be speculated that the present case might represent a distinct variant of HK. Although the first reports suggested that the UV exposure might be involved in the pathogenesis [1], the eruption of our case was unrelated to UV.

Many dermatoses could be considered in the differential diagnosis of DHK including stucco keratosis, verruca plana, lichen nitidus, Darier's disease, and guttate psoriasis. The diagnosis of DHK was established based on the exclusion of the differential diagnosis regarding the clinical and histological features.

A few treatment modalities were tried for the HK in the previous reports including tretinoin, propylene glycol, salicylic acid gel, and cryosurgery without significant improvement [1, 2]. One exception is that tretinoin gel and salicylic acid ointment provided marked improvement in the case of the localized HK reported by Kokturk et al. [3]. We tried to use oral acitretin for the treatment of the present case considering the extensive scaly lesions and the failure of the topical therapies reported before. The significant response to the retinoid therapy leads us to speculate that this disorder may arise from an undetermined pathology of keratinization. However, there is no further evidence to support or disprove this idea.

## 4. Conclusion

DHK is a rarely reported entity with widespread hypopigmented keratotic papules with unknown etiology. Acitretin may be a considerable option for the treatment of HK.

## Figures and Tables

**Table 1:** The general characteristics of the cases of DHK reported in the literature.

Year	Author (ref.)	Age	Gender	Site	Age of onset	Histopathology
1991	Morison et al. [1]	19	Female	Trunk, extremities, and dorsa of the hands and feet	19	Mild orthohyperkeratosis and papillomatosis
1991	Morison et al. [1]	5	Female	Extremities and trunk	5	Biopsy was not performed
2013	Kim et al. [2]	4	Female	Arm, leg, and trunk	2	Hyperkeratosis, acanthosis, decreased melanin content, and number of melanocytes
2013	Kim et al. [2]	5	Female	Arm	0	Same with above
2013	Kim et al. [2]	40	Male	Trunk	33	Biopsy was not performed
2013	Kim et al. [2]	70	Female	Trunk	50	Biopsy was not performed
2017	Present case	21	Male	Trunk, extremities, and face	11	Orthohyperkeratosis, irregular acanthosis, and sporadic papillomatosis
